# Human Gait Recognition: A Single Stream Optimal Deep Learning Features Fusion

**DOI:** 10.3390/s21227584

**Published:** 2021-11-15

**Authors:** Faizan Saleem, Muhammad Attique Khan, Majed Alhaisoni, Usman Tariq, Ammar Armghan, Fayadh Alenezi, Jung-In Choi, Seifedine Kadry

**Affiliations:** 1 Department of Computer Science, HITEC University Taxila, Taxila 47080, Pakistan; faiziminhas12@gmail.com (F.S.); attique.khan@hitecuni.edu.pk (M.A.K.); 2 College of Computer Science and Engineering, University of Ha’il, Ha’il 55211, Saudi Arabia; majed.alhaisoni@gmail.com; 3 College of Computer Engineering and Science, Prince Sattam Bin Abdulaziz University, Al-Kharaj 11942, Saudi Arabia; u.tariq@psau.edu.sa; 4 Department of Electrical Engineering, College of Engineering, Jouf University, Sakakah 72388, Saudi Arabia; aarmghan@ju.edu.sa (A.A.); fshenezi@ju.edu.sa (F.A.); 5 Department of Applied Artificial Intelligence, Ajou University, Suwon 16499, Korea; 6 Faculty of Applied Computing and Technology, Noroff University College, 4608 Kristiansand, Norway; seifedine.kadry@noroff.no

**Keywords:** gait recognition, biometric, data augmentation, deep learning, features optimization, features fusion

## Abstract

Human Gait Recognition (HGR) is a biometric technique that has been utilized for security purposes for the last decade. The performance of gait recognition can be influenced by various factors such as wearing clothes, carrying a bag, and the walking surfaces. Furthermore, identification from differing views is a significant difficulty in HGR. Many techniques have been introduced in the literature for HGR using conventional and deep learning techniques. However, the traditional methods are not suitable for large datasets. Therefore, a new framework is proposed for human gait recognition using deep learning and best feature selection. The proposed framework includes data augmentation, feature extraction, feature selection, feature fusion, and classification. In the augmentation step, three flip operations were used. In the feature extraction step, two pre-trained models were employed, Inception-ResNet-V2 and NASNet Mobile. Both models were fine-tuned and trained using transfer learning on the CASIA B gait dataset. The features of the selected deep models were optimized using a modified three-step whale optimization algorithm and the best features were chosen. The selected best features were fused using the modified mean absolute deviation extended serial fusion (MDeSF) approach. Then, the final classification was performed using several classification algorithms. The experimental process was conducted on the entire CASIA B dataset and achieved an average accuracy of 89.0. Comparison with existing techniques showed an improvement in accuracy, recall rate, and computational time.

## 1. Introduction

Human gait recognition has become an active research area in the past decade in computer vision [1,2]. Biometrics is an important application of gait recognition, employed in many industrial areas such surveillance and healthcare systems [3,4]. Despite the specific characteristics of gait features, several factors influence gait identification, including camera perspectives, load holding, lighting conditions, clothing variance, walking speed, and shadows under feet [5]. As a result, designing a robust enough framework to solve these obstacles is crucial for accurate gait classification [6]. Moreover, a few biological signals are also useful for gait analysis such as electromyography (EMG) [7,8,9], inertial sensors [10,11], and plantar pressure [12]. Through these methods, the human gait can be analyzed, for example, through muscle movement while walking. Gait recognition algorithms have progressed to the point that they can now be used in a wide range of “real-world” applications, such as video monitoring, crime prevention, and forensic detection [13,14].

The two broad categories of HGR are model-based and model-free approaches [3]. Model-based approaches, such as human body structure and motion models, are used to classify human gait. They use a structural model to characterize a human subject and use underlying mathematical structures such as stick-figure, intertwined pendulum, and ellipse fitting techniques to track different body parts and joint positions over time to define gait [15]. The features of the object silhouette are derived from the gait period in the model-free method, which is simple to implement due to lower computational costs [16].

Computer vision, a floor sensor, and a watch sensor are three common approaches for detecting gait depending on the background [14]. Using computer vision, cameras are used for video capturing to process the frames’ transformation. In the past two decades, researchers have performed the silhouette extraction process to extract the classical features for gait recognition [17,18]. They extracted the silhouette using GEI [19] and MHI based techniques [20,21]. Moreover, GFHI and Markov Hidden Model-based techniques have also been introduced for gait recognition [22]. These methods are not reliable once the size of the dataset is increased. Moreover, this type of process is time-consuming and takes up a lot of space, which later affects the entire system’s computational time. Further, the main limitation of this process is that if a human silhouette is incorrectly detected, irrelevant features are extracted [23,24]. Researchers have tried to resolve these issues using feature reduction techniques such as principle component analysis (PCA) [25,26], linear discriminant analysis (LDA) [27], and improved PCA [28], among others [29,30].

The most recent research in computer vision applied deep learning for human gait recognition with better recognition accuracy [31]. The researchers used deep learning performance in varying domains such as object identification, disease segmentation, and recognition from photographs and videos [32,33,34,35]. The convolution neural network (CNN) is a type of deep learning, which includes several layers that extract the deep features of each image [36,37]. In a CNN, a few layers are added as an activation function, and a few are utilized for the downsampling [38,39].

The extracted deep features from the raw images also extract some redundant and irrelevant features. These types of features need refinement for better classification accuracy. The researchers introduced feature selection techniques to handle irrelevant features for classification purposes [40]. Feature selection (FS) is an active research area in machine learning currently [41]. The purpose of the feature selection step is to refine the high dimensional feature vector, which contains duplicate and unnecessary features for classification. The primary goal of FS is to eliminate redundant and unnecessary characteristics from the original feature vector to reduce its dimensionality and to choose the single most vital point for higher classification accuracy and low computing time. Some of the latest feature selection techniques are an entropy-based approach, a Genetic Algorithm based approach, and a grasshopper based approach, among others [42,43].

**Problem Statement:** The key problems this research article addresses are:

- Recognizing gait in various circumstances, such as changing view angles and walking styles while still wearing a coat and bag, is a difficult challenge.
- Different subjects have their distinct gait; however, some of the subjects have a very similar gait. This issue misleads the correct classification and degrades the system’s performance.
- Some researchers follow the two step process for gait recognition, such as subject detection and then classification. However, this first step is not a guarantee for accurate recognition of human gait due to incorrect subject detection. Also, two-step process increases the computational time.
- Variation in the style of clothings such as a long coat, half shirt, skirt, regular pants, coats, and so on. In these styles, it is not easy to extract the rich features for further classification.
- The irrelevant features information which is extracted from the original frames is an impact on the system accuracy and computational time.

**Contributions:** A new framework is proposed using deep learning and selection of optimal features for human gait recognition to resolve the above-listed issues. The significant contributions are as follows:

- Modified two pre-trained deep learning models on CASIA B dataset and performed transfer learning. After the transfer learning, features are extracted from the average pool layer.
- Features are fused using a modified mean absolute deviation extended serial fusion (MDeSF) approach.
- The best features are selected using a three-step Improved Whale Optimization algorithm.

The rest of the manuscript is organized as follows: Recent gait recognition techniques are discussed in Section 2. An explanation of the dataset is provided in Section 3. Section 4 presents the proposed methodology. The results of the proposed method are discussed in Section 5. Finally, we conclude.

## 2. Related Work

Deep learning showed much success in the area of machine learning in the past two decades. Well known applications of deep learning include visual surveillance, biometrics, and medicine, among others [44,45]. Biometrics is an important application and much research attends to this field [46]. In the biometric field, gait recognition is an important research area and several techniques using deep learning have been introduced [47].

Davarzani et al. [48] presented a system for human gait detection based on deep learning. They implemented three models at the initial stage such as linear regression, artificial neural network (ANN), and LSTM. Later on, the performance of each model was combined for the final detection. The experimental process was conducted on each model using publically available datasets. The results showed that the ANN performed well as compared to linear regression and LSTM. Asif et al. [3] presented an end-to-end system based on pre-trained convolutional neural network features and best features selection. They used deep learning for the deep features extraction, and then best features were selected for the final classification. Three different angles of the CASIA B dataset were used in the experimental process, 18, 36, and 54. The results showed that the precision and recall rate of the presented method was better than the current state-of-the-art methods.

Anusha et al. [49] described a Modified Local Optimal Oriented Pattern Binary (MLOOPB) descriptor for clothing-invariant HGR. The MLOOPB descriptors were the extension of LOOP descriptors. From MLOOPB, different types of feature vectors such as the histogram and horizontal width variables were extracted. Then, the extracted features were reduced using a new approach, and classification was performed. The experimental process was conducted on the OU-ISIR B treadmill gait dataset and the CASIA B gait dataset and showed improved accuracy over the compared techniques. Habiba et al. [1] presented an integrated framework for HGR using deep features and a hybrid feature selection method. They employed pre-trained models for deep features extraction. The extracted features were selected using a fuzzy entropy controlled skewness (FEcS) approach. Four popular datasets were used for the experimental process, CASIA A, CASIA B, CASIA C, and AVAMVG and showed better performance than existing techniques.

Kooksung et al. [50] presented an automated feature extraction approach using a recurrent neural network (RNN)-based auto encoder (AE) for skeleton-based abnormal gait identification. They introduced two models named gated recurrent unit (GRU)-based AE (GRU AE) and an LSTM-based AE (LSTM AE). Using these two models, they extracted deep features and performed identification. Based on the experimental process, it was noted that the LSTM AE outperformed the GRU AE in terms of efficiency for gait identification. Anusha et al. [51] extracted low-dimensional features such as gradient, spatial, and texture for gait recognition. They used a nine-cell gait gradient magnitude image to compute a histogram of oriented gradients. After that, Haralick texture descriptors were extracted and concatenated for final recognition. The experiment was carried out on five gait datasets and obtained better accuracy than the existing compared techniques.

Khan et al. [5] presented a cross-view gait identification using a view-invariant gait representation model. They trained a deep fully connected neural network to convert gait descriptors from different points of view into a single canonical view. Later, the single canonical view descriptors were used for the experimental process. The experimental process was conducted on the OU-ISIR large population and CASIA-B datasets and showed that the presented cross-view gait recognition method had improved accuracy. Elharrouss et al. [52] presented a deep learning structure for HGR. They used multitask CNN architectures and extracted gait energy images (GEIs). Based on the GEI, features were extracted that were later classified using CNN. The experimental process was conducted on the CASIA B, OU-ISIR, and OU-MVLP datasets. The results on the selected datasets were better than current methods.

A few other studies were also conducted for HGR, such as PoseGait and the CNN model [13], a multichannel CNN architecture [38], GEI and CNN architecture [53], and RCNN based gait recognition [54], among others [55]. The methods mentioned focused on the middle step, for example, GEI, handcrafted features, and CNN. They did not focus on the important steps of the fusion of features from different models and selecting the best features for better computational time. This article proposes a new framework using deep learning features selection and multisource features fusion for HGR.

## 3. Dataset Detail

**Figure 1:**
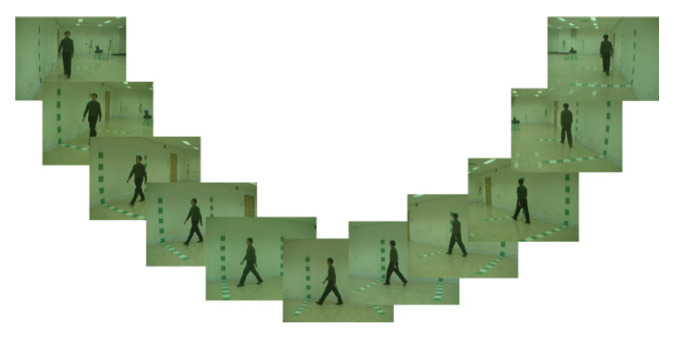
Sample video frames from the CASIA B dataset.

**CASIA B Dataset:** CASIA B [56] is commonly implemented to identify patterns in multiple viewing of various clothing conditions, also known as a multiview home dataset [57]. This database includes 124 headings with gait sequences registered from 11 different angles of 0, 18, 36, 54, 72, 90, 108, 126, 144, 162, and 180 degrees, with three different styles of walking sequences, including walking with a backpack, wearing a hat, and a regular walking sequence. Figure 1 shows sample frames from the CASIA B dataset. Each video of the gait sequence is captured at a rate of 25 frames per second with a resolution of 352 × 240 pixels. In this work, all angles were selected for the experimental process. 

## 4. Materials and Methods

**Figure 2:**
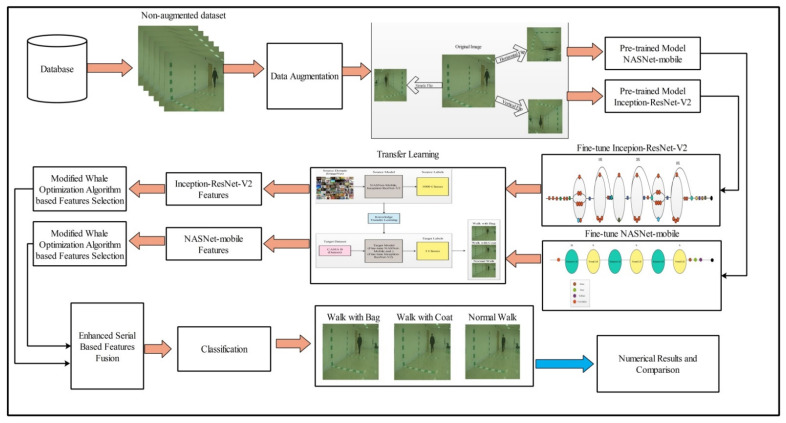
The main architecture of the proposed methodology for HGR.

A new framework was proposed for human gait recognition, including many steps such as data augmentation, feature extraction, feature fusion, and feature selection. The architecture of the proposed framework is shown in Figure 2. In the augmentation step, three flip operations were used. In the feature extraction step, two pre-trained models were used named Inception-ResNet-V2 and NASNet Mobile. Both models were fine-tuned and trained using transfer learning on the CASIA B dataset (three classes; walk with bag, walk with coat, and basic walk for five different angles 18, 36, 54, 72, and 90, extracting the features). The features of the selected deep models were optimized using a modified three-step whale optimization algorithm and the best features were selected. The selected best features were fused using the modified mean absolute deviation extended serial fusion (MDeSF) approach. Finally, the fused vector was classified using several machine learning classifiers. The details of each substep is given below.

### 4.1. Data Augmentation

The process of expanding the quantity of data utilized for training a model is known as data augmentation [58]. When preparing a machine learning model, it works as a regularizer and helps to minimize overfitting. Some of the main techniques used in data augmentation in deep learning are: the flip operation, rotation operations, scale operation, crop operation, translation operation, and Gaussian noise. In this proposed framework, data augmentation was performed using translation operations such as simple translation, horizontal translation, and vertical translation. The primary purpose of data augmentation is to balance the data and improve accuracy. In this work, the CASIA B dataset was utilized for the evaluation process [56]. This dataset is available in the form of videos having three different classes—normal walk, walk carrying a bag, and walk wearing a coat. This dataset was captured from 11 different angles with a total of 124 subjects. The entire dataset was considered and split into training and testing, with a ratio of 50:50 instead of 70:30. This meant that 50% of the videos of each class were selected for training, and the remaining 50% were utilized for testing. Each angle of this dataset consisted of three classes, and each class contained a different number of video frames. Some classes had 2000 frames, and some had 2500. To balance the numbers of frames, translation operations were performed. By employing the translation operations, we increased the numbers of frames from 2500 to 6000 frames.

#### 4.1.1. Left and Right Horizontal Translation

Mathematically, these operations were formulated as follows:

(1) x=f(z−a)

where f(z) is the original function and a is a constant added or removed to create a horizontal change in the constant of function. If the function a is positive, it is moved to the right, and if the function a is negative, it is transferred to the left. To examine horizontal translations, the identical fundamental quadratic function is utilized. We have a basic quadratic equation:

(2) $$x=z^{2}$$

By shifting the function to the left by two, the following equation is employed:

(3) x=f(z+a)

(4) $$={\left(x+2\right)}^{2}$$

Here, a is a constant. By shifting the function to the right by two, the following equation is employed:

(5) x=f(z−a)

(6) $$={\left(x-2\right)}^{2}$$

Vertical translation shifts the function up or down. It is defined as follows:

(7) x=f(z)+a

where f(z) is the function and a is the constant of this function to enable translation. Consider the quadratic equation:

(8) $$x=z^{2}$$

#### 4.1.2. Up and Down Vertical Translation

Then, the vertical translation can be defined as follows:

(9) $$x=z^{2}+2$$

(10) $$x=z^{2}-2$$

**Figure 3:**
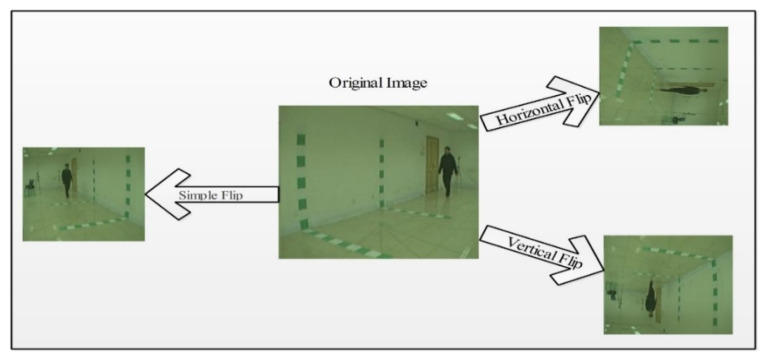
Output of the data augmentation step.

Visually, the output of these operations is shown in Figure 3. The resultant images were later utilized to train the CNN models.

### 4.2. Convolutional Neural Networks (CNN)

**Figure 4:**
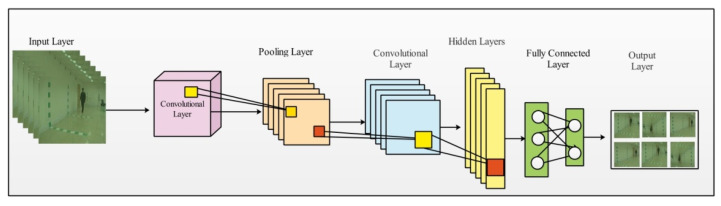
Convolutional neural network for the classification task.

The convolutional neural network (CNN) is a type of deep learning that includes multiple layers that surpasses classical methods such as object classification, among others [46,59]. The layers of CNN were formed by a convolutional operation followed by an activation layer (ReLu). Other layers are included in a CNN, such as pooling, normalization, fully connected, average pool, and Softmax. Figure 4 illustrates a simple CNN model for the classification task. 

#### 4.2.1. Convolutional Layer

Initially, the selected dataset frames were utilized as an input to the convolutional layer. A convolutional layer connects a set of M filters to a group of T channels and size A×B in comparison to a mini-batch of X Images with T channels and size Height×widhth. The filter elements are denoted by $E_{w,x,y,z}$ and image elements denoted by $F_{g,h,i,j\;}$, then the formulation of the convolutional operation is defined as follows:

(11) $$Y_{g,w,i,j}=\overset{T}{\underset{c=1}{\sum }}\overset{R}{\underset{u=1}{\sum }}\overset{S}{\underset{v=1}{\sum }}F_{g,h,i,j+y,j+z\;}E_{w,x,y,z}$$

And the output of a whole image/filter combination can be written as:

(12) $$Y_{g,w}=\overset{T}{\underset{c=1}{\sum }}F_{g,h}*\;E_{w,x}$$

where ∗ show the two-dimensional correlation.

#### 4.2.2. ReLU Layer

The goal of ReLU is to augment the CNN’s nonlinearity. Using this layer, the negative weights values were converted to zero. The rest of the positive values will be considered the same for the next process.

(13) ReLU(A)=max{0,a}

The weights were adjusted with the updating rule by employing the following equation:

(14) $$Wgt^{*}=w+\delta \frac{\gamma F^{2}}{\gamma w}$$

where the new weight is $Wgt^{*}$ with current value w, and the learning rate is γ.

#### 4.2.3. Pooling Layer

The pooling layer is mostly employed for the downsampling of a feature map. Normally, three types of pooling operations are employed: maximum pooling, average pooling, and minimum pooling. Mathematically, these operations are defined as follows:

(15) $$PL^{i}=Maxpool{\left(PL\right)}^{i-1}$$

Maxpool represents the max pooling, and i is the input matrix. 

#### 4.2.4. Batch Normalization

Normalization methods can reduce the training time of the selected model by a large proportion [60]. Batch normalization is a technique that normalizes network activity over a defined mini-batch size. For the mini-batch size, mean and variance were computed. Then the mean was subtracted, and the feature was divided by its standard mini-batch deviation.

(16) $$\partial_{A}\leftarrow \frac{1}{n}\overset{n}{\underset{i=1}{\sum }}x_{i}$$

(17) $$\omega_{A}^{2}\leftarrow \frac{1}{n}\overset{n}{\underset{i=1}{\sum }}{\left(x_{i}-\partial_{A}\right)}^{2}$$

where $\partial_{A}$ represents the mini-batch mean, and $\omega_{A}^{2}$ is the mini-batch variance. 

#### 4.2.5. Fully Connected Layer

The CNN extracted feature was concatenated in the fully connected layer $f_{full}=f_{CNN}$. Then, the classification result was produced using a softmax operation as described by the following formulation:

(18) $$p=Softmax\left(f_{full}\;*\;w_{p}+b_{p}\right)$$

where p is the output sample, the weight matrix of the output layer is denoted by $w_{p\;}$ and the output bias is denoted by $b_{p}$. The fully connected Layer (FC) functions on a flattened input, where all neurons are linked to each input.

### 4.3. Transfer Learning

**Figure 5:**
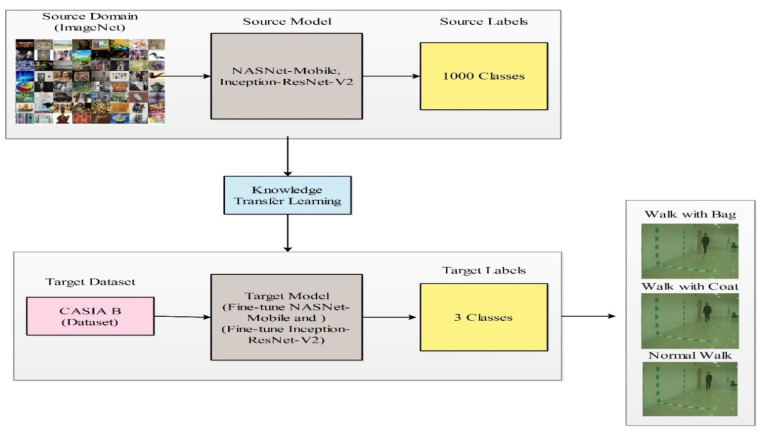
Process of transfer learning for HGR.

Transfer learning (TL) is a process of reusing a pre-trained model for another task. Originally, the pre-trained deep models were trained on the ImageNet dataset, which included 1000 object classes. Consider a domain B made up of the following components: a feature space W and a marginal probability distribution P(W), where M={m1,……,mn}∈X. The next specific domain is W=M,P(M). This task consists of two main components, label space L and objective predictive functions f: A→B, in the presence of a source domain Xs and learning task Ys_,_ a target domain $X_{t}$ and learning task $Y_{t}$, where Xs≠ $X_{t}$ and $Ys\;\neq \;Y_{t}$. The goal of transfer learning is to aid in the learning of the target predictive function. This process is illustrated in Figure 5. 

In this work, TL was carried out on two modified pre-trained models Inception-ResNet-V2 and NasNet-model. Both models were trained through TL on the CASIA B gait dataset, where several parameters were utilized, as shown in Table 1. These parameters were initialized based on the size of the dataset and performance of the Desktop computer. However, these parameters can be changed according to the learning environment. After training, these models were saved and utilized for features extraction. 

### 4.4. Deep Features of Modified Inception-ResNetV2

**Figure 6:**
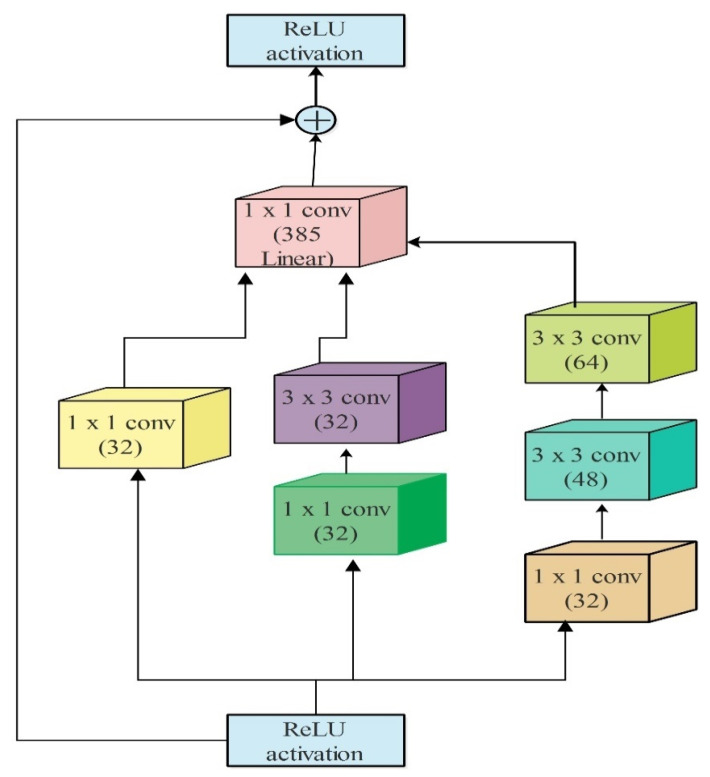
Interior block models of inception-ResNet-V2.

Inception-ResNetV2 is a CNN model originally trained on the ImageNet dataset, consisting of 164 layers. The network is strong and has learned feature representations for a wide range of images types. This network accepts an input of 299×299 and hybridization of two recently developed networks, residual links, and the latest architectural version. There are series of filters such as 1×1, 3×3, 5×5, etc., fused with each branch concatenation. The split-transformation-mixing structure of the initiation module is seen in its thick layers as a strong representation. Figure 6 depicts the inner architecture of Inception-ResNet-v2. The residual connections enable the training of increasingly deeper neural networks, resulting in an even more outstanding performance. In this work, this model was fine-tuned and trained using TL. The detail of TL is given in Section 4.3. Features were extracted from the GAP layer and a feature vector of dimension N×1536  was obtained, where N represented the number of images used for the testing purpose. 

### 4.5. NASNet-Mobile

NASNet-Mobile is a scalable CNN (built by neural architecture searches) architecture consisting of fundamental building blocks (cells) improved by reinforcement learning. A cell comprises a few operations (many separation convolutions and pooling) and is repeated numerous times depending on the needed network capacity. The Mobile version contains 12 cells with 5.3 million parameters and a multiply-accumulated total of 564 million (MACs) [61]. The fundamental element of NASNet incorporates a regularization technology from Scheduled DropPath, which substantially increases model generalization. In the fine-tuning process, new layers were added and the entire network was trained on the Gait dataset (CASIA B). After the training, features were extracted from the convolutional layer, and a feature vector of dimension N×1056 was obtained. 

### 4.6. Novelty 1: Feature Selection

This work used a modified whale optimizer algorithm (WOA) for the best features selection. The modified whale optimization algorithm works in three phases:

- Phase of Exploitation (circle technique of attack of prey/bubble-net)
- Phase of Exploration (searching prey)
- Define a new activation function to check the redundant features

#### 4.6.1. Phase of Exploitation (Circle Technique of Attack of Prey/Bubble-Net)

The following formulation depicts the movement of a whale near prey [62]:

(19) $$\vec{A}=\left|\vec{B}\cdot Z^{*}\;\left(T\right)-\vec{Z}\left(T\right)\right|$$

(20) $$\vec{Z}\left(T+1\right)=\vec{Z^{*}}\left(T\right)-\vec{C}-\vec{A}$$

where T shows the current iteration, $Z^{*}$ shows the best solution obtained, Z shows the best solution, || offers the absolute value, and (·) represents a multiplication symbol. The coefficient vectors A and C are discussed in the following equations:

(21) $$\vec{C}=\;2\;\vec{a}\cdot \;\vec{r}-a$$

(22) $$\vec{A}=2\cdot \vec{r}$$

where a decreases linearly from 2 to 0, and r is a random vector in the range [0,1]. According to the above equation, solutions are based on the position of the most known solution to update their locations.

(23) $$a=2-t\frac{2}{MaxIter}$$

where T is the number of iterations, and MaxIter is the maximum number of iterations permitted. The current and best (leading) solutions are formulated as follows:

(24) $$\vec{Z}\;\left(T+1\right)=\overset{´}{K}\cdot e^{dl}\cdot \cos\left(2\pi l\right)+\vec{Z^{*}}\left(T\right)$$

where the distance is defined by ($\vec{A}=\left|\;\vec{Z^{*}}\;\left(T\right)-\vec{Z}\left(T\right)\right|$), d represents the spiral shape, and l determines the random number [−1,1].

#### 4.6.2. Phase of Exploration (Searching Prey)

Instead of requiring solutions to search randomly based on the location of the best solution, a randomly chosen solution is utilized to update the position [62]. Consequently, a C vector is utilized to control a solution, which is deviating from the best-known search agent with random values greater or less than one. The following formulation can be utilized for this process.

(25) $$\vec{C}=\left|\vec{C}\;\cdot {\vec{Z}}_{\;rand}-\vec{Z\;}\right|$$

(26) $$\vec{Z}\left(T+1\right)={\vec{Z}}_{\;rand}-\vec{C}\cdot \vec{A}$$

where ${\vec{Z}}_{\;rand}$ is a randomly selected whale from the existing population. 

#### 4.6.3. Phase of Final Selection

In the next step, the existing algorithm was modified in the final selection phase. The mean deviation was computed by $\vec{Z}$ and multiplied with the highest feature value of the entire selected vector. After that, we searched for the redundant features and each redundant feature was replaced with the product value multiplied by the i−1 feature. Mathematically, this process is defined as follows:

(27) $$Rd\left(k\right)=Search\left(i,j\right)\;Rep\left(k\right)=Val\times \left(i-1\right)\;MAD=\frac{\sum \left|\phi -\bar{\phi }\right|}{N}\;H=Max\left(\vec{Z}\right)$$

(28) Val=MAD×H

The selected best features are fused in a later step using the proposed fusion approach. In this work, the size of the final selected features was N×560 and N×766; however, this size can be updated according to the selected dataset dimension. The berief description is also added in Algorithm 1.

**Algorithm 1** Modified Whale Optimization for Best Features Selection
*1.* 
*Initial population generation* $x_{i}\left(i=1,2,\ldots ,n\right)$

*2.* 
*Calculate each solution’s objective value*
*3.* $x_{*}$ = *best solution**4.* 

while(t<Max_Iteration)

*5.* 

    for *each solution*
*6.* 

           Update a, A, C, l, and p

*7.*       if 1(p<0.5)*8.*                     if 2(|A|<1)*9.*       *To update the current solution’s location, use Equation (20).**10.*                     else if 2(|A|<1)*11.*        *Choose a solution at random* ${\vec{Z}}_{rand}$*12.*           *Use Equation (25)**13.*                    end if 2*14.*                    else if 1(p≥0.5)*15.*      *Update the current search position using Equation (24)**16.*                   end if 1*17.*            end for*18.* 
*Verify that some solution exceeds and modifies the search area.*
*19.* 
*Determine the fitness of each solution.*
*20.* 
*Update* $X^{*}$ *if a better solution becomes available.*
*21.*            t=t+1*22.*     end while*23.*     $return\;X^{*}$*24.* 
*Perform Search Function* Rd(k)

*25.*      *-Find Redundant Features **26.*      *-Replace with* Rep(k)*27.*      *-Again determine the fitness**28.*   $return\;X^{*}$


### 4.7. Novelty 2: Feature Fusion

Features fusion represents the combination of features from the different layers or branches [63,64]. In this work, a modified features fusion approach was employed, the mean absolute deviation extended serial fusion (MDeSF). This approach was implemented in two steps. In the first step, features of both vectors were sorted into the descending order and then serially fused in one matrix. In the second step, the mean absolute deviation was computed and a threshold function defined. Based on the threshold function, the final features were added in the fused vector.

Assume X and Y are two pattern sample space-defined feature spaces. The corresponding two feature vectors α∈X and β∈Y for an arbitrary sample were ȴ∈Ȗ. The feature vectors α and β were sorted into descending order using the following expression:

(29) α(i)=Desc(α),  β(j)=Desc(β)

The serial combination feature vector is described as:

(30) $$Ƒ=\left(\begin{array}{c} \alpha \left(i\right)\\ \beta \left(j\right) \end{array}\right)$$

If α is an a-dimensional feature vector and β is the b-dimensional, then the combined serial function ϕ is (a+b) [65]. In the next step, the mean absolute deviation was computed from the obtained feature vector Ƒ, and a threshold function was designed as follows:

(31) $$MAD=\frac{\sum \left|\phi -\bar{\phi }\right|}{N}$$

(32) $$Thd=\{\begin{array}{c} \psi \left(k\right)\;\;\;\;\;for\;\;\;\phi \geq MAD\\ Ignore,\;\;\;\;\;\;Elsewhere \end{array}$$

The above threshold function represented the features of the originally fused vector ϕ compared with the MAD value, those which are greater or higher are added in the final fused vector ψ(k). The features, which did not fulfill this criterion, were removed from the fusion list. The resultant feature vector was finally classified using machine learning algorithms.

## 5. Results

### 5.1. Experimental Setup

The proposed method was evaluated on a publicly available dataset, CASIA B. Details of this dataset are given in Section 3. Seven experiments were performed in this work with two models: (i) Inception-Resnet-V2 and (ii) Nasnet Mobile. Initially, the experiments were conducted on deep individual models using a non-augmented dataset, and an augmented dataset was then employed. The main purpose of these experiments was to analyze the performance of the augmentation process. In the next experiment, a feature selection algorithm was applied on both deep models and finally, fusion was performed. The classifiers used in the experiments were C-SVM, Q-SVM, SD, MG-SVM, F-KNN, LD, L-SVM, S-KNN, W-KNN, M-KNN, and C-KNN. For each classifier, we included the recall rate, F1-Score, accuracy, and computational time. The proposed method was implemented inn MATLAB 2020b using a personal computer with a 64-bit operating system, 16 GB of RAM, and an 8 GB graphics card.

### 5.2. Results

The results of the proposed method are given in this section. The modified Inception-ResNet V2 model was trained on a non-augmented dataset, and deep features were extracted. The results are given in Table 2. This table shows that the maximum achieved accuracies of Cubic SVM on selected angles were 88.8, 84.6, 85.1, 76.4, and 78.9%. The F1-Score of Cubic SVM was also calculated, and the value of each angle was 88.8, 84.64, 85.1, 76.44, and 79.02%. The performance of Cubic SVM was also compared with some other classification methods such as QSVM, MG SVM, and SD and showed that the performance of Cubic SVM was better in terms of recall rate, F1-Score, and accuracy. Moreover, each classifier noted the computational time showing that the QSVM executed faster than other classifiers. 

Table 3 presents the results of the modified NasNet Mobile on selected angles of the non-augmented CASIA B dataset. This table shows the maximum accuracies of 87.0%, 83.6%, 80.4%, 72.3%, and 73.0% for Cubic SVM. The F1-Score values of each angle for Cubic SVM were 86.97%, 83.66%, 80.39%, 72.34%, and 73.04%. Other classifiers were also implemented, such as QSVM, MG SVM, and SD, which showed that the Cubic SVM classifier performed better. However, the computational time of QSVM was better than the Cubic SVM, MGSVM, and SD. Moreover, the accuracy of Inception-ResNet V2 was better than NasNet Mobile, but the computationally modified NasNet Mobile was efficient.

Table 4 presents the results of the modified Inception-ResNet V2 on selected angles of the augmented CASIA B dataset. The Cubic SVM classifier had better classification accuracy of each angle, and the values were 95.6%, 926%, 92.7%, 86.2%, and 87.7%. Compared with the non-augmented results (shown in Table 2), the accuracy after the augmentation process was significantly improved. The values of the F1-Score of each angle for Cubic SVM were 95.58%, 92.62%, 92.7%, 86.16%, and 87.74%. Similarly, the performance of the rest of the classifiers such as QSVM, GSVM, and SD improved. The time was also noted for each classifier for this experiment. Based on the recorded time, the QSVM and Cubic SVM were both efficiently executed. 

Table 5 presents the results of the modified NasNet Mobile on selected angles of the augmented CASIA B dataset. This table shows that the Cubic SVM obtained better accuracy than the other classifiers QSVM, MGSVM, and SD. The obtained accuracies of the Cubic SVM classifier were 94.4%, 92.1%, 90.9%, 83.5%, and 84.8%. Compared with the non-augmented results (shown in Table 3), the accuracy after the augmentation process significantly increased for each angle. The computational time was also noted for each classifier, and the QSVM and Cubic SVM were both efficiently executed.

Table 6 presents the results of the proposed best feature selection algorithm for Inception-ResNet V2 deep features. The augmented CASIA B dataset was utilized for the experimental process. This table presents the best accuracies obtained by Cubic SVM of 95.2%, 92.5%, 91.4%, 85.3%, and 86.4%. Compared with the originally extracted deep features results (shown in Table 4), the accuracy of each classifier for different angles was almost consistent; however, computationally, this step was more efficient. Moreover, the performance of the rest of the classifiers was improved after the employing of a modified WOA. 

Similarly, Table 7 presents the results of the modified WOA for NasNet Mobile. This table shows the Cubic SVM obtained the best accuracies of 94.1%, 91.3%, 90.7%, 82.7%, and 84.0%. Moreover, the comparison of this experiment with Table 5 shows that the accuracies of this experiment were almost consistent; however, the computational time was significantly decreased. This shows the importance of this algorithm.

Finally, the selected best features of modified Inception-ResNet V2 and modified NasNet Mobile were fused using the proposed fusion method. The results of the features fusion are given in Table 8. This table shows the best accuracies were obtained by Cubic SVM with 97.3%, 96.0%, 95.3%, 86.6%, and 89.8%. The F1-Score was computed for each classifier and the best obtained values of 97.24%, 95.99%, 95.28%, 86.61%, and 88.06% were for Cubic SVM. The performance of the fusion process was compared with Table 3, Table 4, Table 5, Table 6 and Table 7; the fusion method improved the recognition accuracy. Moreover, the performance of the rest of the classifiers was also significantly improved. However, the limitation of this step was an increase in the computational time. 

**Figure 7:**
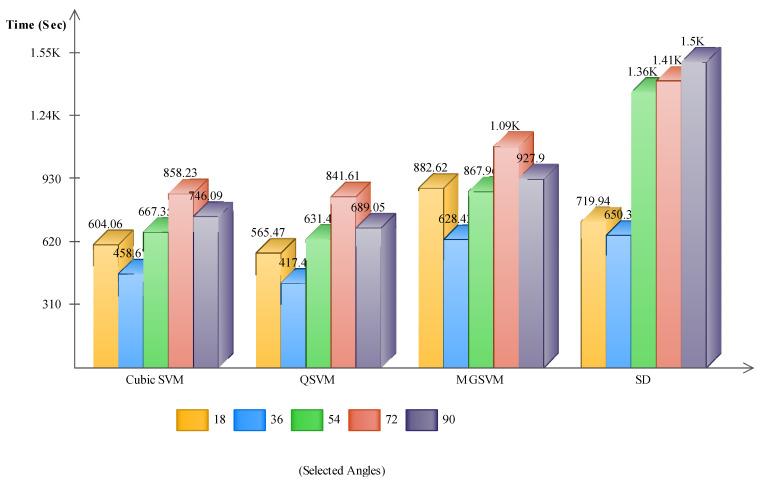
Computational time of the features fusion process using the augmented CASIA B dataset.

**Figure 8:**
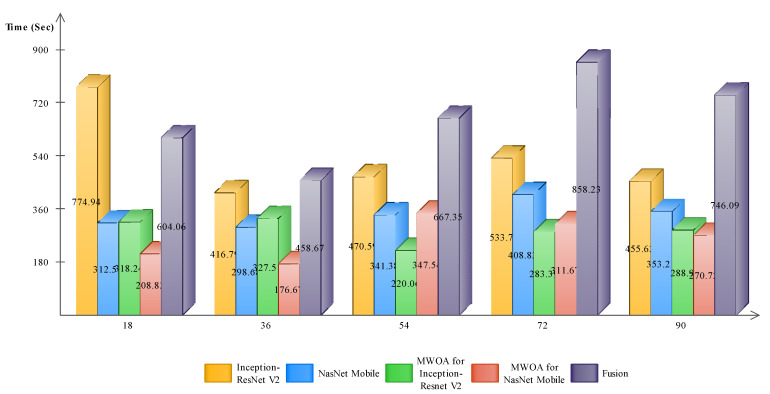
Computational time of Cubic SVM for all five steps.

We briefly discuss the computational time and comparison of the proposed method results with the state-of-the-art (SETA) techniques. Figure 7 shows the time plot of the fusion process and that the Cubic SVM and QSVM executed faster than the MGSVM and SD. Figure 8 shows the computational time of Cubic SVM for all five experiments; the feature selection process consumed less time than the original feature vectors and fused vector.

**Experiments on the Entire CASIA B Dataset:** The proposed framework was finally evaluated on the entire CASIA B dataset and the results are presented in Table 9. Moreover, in this table, a detailed analysis of the proposed method was conducted based on standard error mean ($\sigma_{\bar{x}}$) and confidence interval (CI). The final fusion process was executed 100 times, and we obtained three values- minimum (Min), average (Avg), and maximum (Max). Based on these three values, standard deviation (σ), standard error mean, and CI were calculated. The CI was calculated for the confidence interval 95%, 1.960$\sigma_{\bar{x}}$. On this confidence level, the proposed method accuracy was noted to be consistent after the initialized iterations.

Moreover, a comparison was conducted with the SOTA techniques, shown in Table 10. The references used in this table used the CASIA B dataset for the experimental process. Each method considered all 11 angles for the experimental process. The mean accuracy of each method was added instead of individual angle-based accuracy. The average accuracy was computed based on variations including the normal walk, walk carrying a bag, and walk wearing a coat. Based on the mean values given in Table 10, the proposed method’s accuracy was good. 

## 6. Conclusions

A new framework was proposed for human gait recognition using deep learning. The proposed framework included data augmentation, feature extraction, feature fusion, and feature selection. In the augmentation step, three flip operations were used. The main reason behind data augmentation was to improve the training data, increasing the classification accuracy. In the feature extraction step, two pre-trained models were fine-tuned, Inception-ResNet-V2 and NASNet Mobile. Both models were trained using transfer learning on the CASIA B dataset. The accuracy of these original deep features was not sufficient for the classification; therefore, a feature fusion technique was proposed to improve the classification accuracy. However, this step increased the computational time. A feature selection method was proposed to resolve this problem, which increased the classification accuracy and lowered the computational cost. Overall, the proposed framework achieved better accuracy on the selected dataset as compared with recent techniques. However, the proposed framework also has a few limitations: (i) the modified whale optimization algorithm selected features in two steps; however, there is a chance of reduction in a few important features that later could decrease the classification accuracy, and (ii) the fusion of features using the MAD based threshold function could ignore some important features. In the future, the following steps could be considered:

- Fusion can be performed using various threshold function and then select the best of them based on the accuracy.
- Moreover, OU-MVLP, OU-LP-BAG, TUM-GAID datasets will also be considered for the experimental process.
- Adopt a two-stream approach such as optical flow based and raw images steps for the more accurate recognition accuracy.
- Extract deep features using latest deep models like Efficient Net.

## Figures and Tables

**Table 1:** Configuration parameters of the modified deep learning models.

Epochs	100
Learning Rate	0.05
Mini-Batch Size	64
Learning Method	SGD
WeightLearnRateFactor	20
LearnRateDropPeriod	6

**Table 2:** Gait recognition results using modified inception-ResNet V2 on selected angles of the non-augmented CASIA B dataset.

Classifier	Angles					Performance Measures			
Cubic (SVM)	18	36	54	72	90	Recall Rate (%)	F1-Score	Accuracy (%)	Time (s)
	❖					88.73	88.80	**88.8**	134.74
		❖				84.7	84.64	**84.6**	219.09
			❖			85.1	85.1	**85.1**	234.95
				❖		76.5	76.44	**76.4**	238.18
					❖	79	79.02	**78.9**	207.65
Quadratic (QSVM)	❖					87	87.12	87.1	122.2
		❖				83.5	83.44	83.4	205.2
			❖			83.1	83.06	83.0	213.94
				❖		75.9	75.86	75.8	222.8
					❖	78.5	78.72	78.4	197.3
Medium Gaussian (MGSVM)	❖					81.16	81.44	81.3	150.09
		❖				78.2	78.14	78.1	261.9
			❖			77.23	77.47	77.2	269.01
				❖		72.56	72.57	72.5	268.82
					❖	75.5	76.45	75.6	246.48
Subspace Discriminant (SD)	❖					81.16	81.44	81.3	150.09
		❖				78.2	78.14	78.1	261.9
			❖			77.23	77.47	77.2	269.01
				❖		72.56	72.57	72.5	268.82
					❖	75.5	76.45	75.6	246.48

**Table 3:** Gait recognition results using modified NasNet-Mobile on selected angles of the non-augmented CASIA B dataset.

Classifier	Angles					Performance Measures			
Cubic (SVM)	18	36	54	72	90	Recall Rate (%)	F1-Score	Accuracy (%)	Time (s)
	❖					86.93	86.97	**87.0**	140.32
		❖				83.7	83.66	**83.6**	752.26
			❖			80.43	80.39	**80.4**	165.36
				❖		72.36	72.34	**72.3**	174.19
					❖	73.03	73.04	73.0	161.92
Quadratic (SVM)	❖					85.2	85.29	85.3	136.44
		❖				81.56	81.47	81.4	147.41
			❖			79.26	79.20	79.2	159.89
				❖		71.43	71.41	71.4	162.87
					❖	73.23	73.27	**73.2**	152.06
Medium Gaussian (SVM)	❖					82.56	82.77	82.6	179.14
		❖				78.96	78.94	78.9	171.86
			❖			74.83	74.84	74.7	189.51
				❖		69.6	69.62	69.5	195.77
					❖	70.1	70.77	70.2	188.1
Subspace Discriminant	❖					81.8	81.86	81.9	244.1
		❖				75.3	75.24	75.2	231.83
			❖			74.7	74.59	74.5	246.87
				❖		69.23	69.17	69.1	245.52
					❖	70.53	70.59	70.4	358.05

**Table 4:** Gait recognition results using modified Inception-ResNet V2 on selected angles of the augmented CASIA B dataset.

Classifier	Angles					Performance Measures			
Cubic (SVM)	18	36	54	72	90	Recall Rate (%)	F1-Score	Accuracy (%)	Time (s)
	❖					95.56	95.58	**95.6**	774.94
		❖				92.6	92.62	**92.6**	416.79
			❖			92.7	92.7	**92.7**	470.59
				❖		86.16	86.16	**86.2**	533.7
					❖	87.76	87.74	**87.7**	455.63
Quadratic (SVM)	❖					93.73	93.74	93.7	613.87
		❖				89.86	89.88	89.9	405.87
			❖			90.63	90.61	90.6	466.01
				❖		83.46	83.42	83.5	546.39
					❖	84.23	84.21	84.2	453.3
Medium Gaussian (SVM)	❖					88.93	89.09	88.9	877.84
		❖				85.56	85.59	85.6	553.11
			❖			86.26	86.30	86.3	617.03
				❖		79.93	79.89	79.9	645.77
					❖	81.23	81.21	81.2	553.25
Subspace Discriminant	❖					89.1	89.14	89.1	1101.9
		❖				86.4	86.4	86.4	814.27
			❖			86.36	86.36	86.4	696.59
				❖		79.56	79.49	79.6	708.82
					❖	81.83	81.77	81.8	860.23

**Table 5:** Gait recognition results using modified NasNet Mobile on selected angles of the augmented CASIA B dataset.

Classifier	Angles					Performance Measures			
Cubic (SVM)	18	36	54	72	90	Recall Rate (%)	F1-Score	Accuracy (%)	Time (s)
	❖					94.43	94.46	**94.4**	312.57
		❖				92.13	92.13	**92.1**	298.68
			❖			90.9	90.9	**90.9**	341.38
				❖		83.5	83.5	**83.5**	408.83
					❖	84.8	84.78	**84.8**	353.21
Quadratic (SVM)	❖					92.43	92.43	92.4	318.57
		❖				89.76	89.76	89.7	311.06
			❖			87.83	87.83	87.8	341.79
				❖		80.8	80.74	80.8	394.58
					❖	81.73	81.71	81.7	336.79
Medium Gaussian (SVM)	❖					90.65	90.70	90.6	408.38
		❖				87.2	87.21	87.2	384.16
			❖			84.3	84.3	84.1	424.26
				❖		77.6	77.6	77.6	484.31
					❖	78.83	78.86	78.9	408.07
Subspace Discriminant	❖					82.9	82.92	82.9	372.4
		❖				80.13	80.14	80.2	461.67
			❖			79.36	79.34	79.4	392.91
				❖		77.63	75.54	73.6	367.88
					❖	75.46	75.42	75.5	345.69

**Table 6:** Gait recognition results using modified WOA for Inception-ResNet V2 on the augmented CASIA B dataset.

Classifier	Angles					Performance Measures			
Cubic (SVM)	18	36	54	72	90	Recall Rate (%)	F1- Score	Accuracy (%)	Time (s)
	❖					95.16	95.16	**95.2**	318.24
		❖				92.53	92.53	**92.5**	327.51
			❖			91.36	91.36	**91.4**	220.06
				❖		85.3	85.3	**85.3**	283.39
					❖	86.33	86.31	**86.4**	288.9
Quadratic (SVM)	❖					92.93	92.96	92.9	319
		❖				89.43	89.5	89.5	327.05
			❖			88.9	88.88	88.9	213.95
				❖		83.06	83.02	83.1	273.6
					❖	83.36	83.32	83.4	284.15
Medium Gaussian (SVM)	❖					88.8	88.96	88.8	419.4
		❖				86	86.01	86.0	416.31
			❖			85.1	85.14	85.1	251.64
				❖		80	79.96	80.0	309.81
					❖	80.73	80.73	80.7	350.5
Subspace Discriminant	❖					85.23	85.29	85.2	435.82
		❖				83.63	83.59	83.6	380.47
			❖			77.66	77.66	77.7	153.97
				❖		75.7	75.59	75.7	221.19
					❖	78.83	78.77	78.8	331.38

**Table 7:** Gait recognition results using modified WOA for NasNet Mobile on the augmented CASIA B dataset.

Classifier	Angles					Performance Measures			
Cubic (SVM)	18	36	54	72	90	Recall Rate (%)	F1- Score	Accuracy (%)	Time (s)
	❖					94.1	94.1	**94.1**	208.83
		❖				91.33	91.33	**91.3**	176.67
			❖			90.7	90.68	**90.7**	347.54
				❖		82.66	82.69	**82.7**	311.67
					❖	83.96	83.98	**84.0**	270.72
Quadratic (SVM)	❖					91.73	91.76	91.7	213.91
		❖				88.23	88.24	88.2	184.07
			❖			87.83	87.81	87.8	341.09
				❖		79.36	79.32	79.4	304.87
					❖	80.83	80.83	80.8	254.24
Medium Gaussian (SVM)	❖					90	90.11	90.0	257.75
		❖				86.06	86.09	86.1	198.9
			❖			83.6	83.67	83.8	415.73
				❖		77.1	77.1	77.1	369.89
					❖	77.83	77.8	77.8	294.81
Subspace Discriminant	❖					78.6	78.62	78.6	193.54
		❖				73.66	73.66	73.7	146.74
			❖			78.2	78.2	78.2	357.09
				❖		71.5	75.42	71.5	258.36
					❖	72.13	72.16	72.2	205.73

**Table 8:** Proposed gait recognition results using the selected best features’ fusion.

Classifier	Angles					Performance Measures			
Cubic (SVM)	18	36	54	72	90	Recall Rate (%)	F1- Score	Accuracy (%)	Time (s)
	❖					97.23	97.24	**97.3**	604.06
		❖				96.03	95.99	**96.0**	458.67
			❖			95.3	95.28	**95.3**	667.35
				❖		86.63	86.61	**86.6**	858.23
					❖	86.43	88.06	**89.8**	746.09
Quadratic (SVM)	❖					95.96	95.98	96	565.47
		❖				95.06	95.08	95.1	417.4
			❖			93.4	93.44	93.4	631.4
				❖		84.1	84.04	84.1	841.61
					❖	87.36	87.34	87.4	689.05
Medium Gaussian (SVM)	❖					94.03	94.11	94.1	882.62
		❖				93.03	93.03	93	628.42
			❖			91.5	91.53	91.5	867.96
				❖		81.86	81.80	81.9	1089.3
					❖	85.73	85.73	85.7	927.9
Subspace Discriminant	❖					94.66	94.68	94.7	719.94
		❖				93.16	93.16	93.1	650.3
			❖			92.23	92.24	92.2	1356.5
				❖		82.8	82.74	82.8	1411.7
					❖	87.26	87.23	87.2	1504.8

**Table 9:** Proposed gait recognition results using best-selected features fusion.

${\mathrm{Angle}}^{{}^{\circ}}$	Min (%)	Avg (%)	Max (%)	σ	$\sigma_{\bar{x}}$	CI
0	71.4	72.3	73.2	0.90	0.6363	72.3 ± 1.247 (±1.73%)
18	96.1	96.70	97.3	0.60	0.4242	96.7 ± 0.832 (±0.86%)
36	95.3	95.65	96.0	0.35	0.2474	95.65 ± 0.485 (±0.51%)
54	93.8	94.55	95.3	0.75	0.5303	94.55 ± 1.039 (±1.10%)
72	85.2	85.90	86.6	0.70	0.4949	85.9 ± 0.97 (±1.13%)
90	88.5	89.15	89.8	0.65	0.4596	89.15 ± 0.901 (±1.10%)
108	93.1	93.85	94.6	0.75	0.5303	93.85 ±1.039 (±1.11%)
126	91.8	92.8	93.8	1.0	0.7071	92.8 ± 1.386 (±1.49%)
144	78.6	80.0	81.4	1.4	0.9899	80 ± 1.94 (±2.43%)
162	88.4	89.6	90.8	1.2	0.8485	89.6 ± 1.663 (±1.86%)
180	77.6	78.3	80.3	0.75	0.5303	78.35 ± 1.039 (±1.33%)

**Table 10:** Comparison of proposed method with STOA techniques.

Reference	Angle	Mean Accuracy (%)
Chao et al. [57], 2019	All 11 angles with three variations	84.20
Wu et al. [66], 2016	All 11 angles with three variations	73.49
Mehmood et al. [3], 2020	18, 36, 54	94.26
**Proposed**	All 11 angles with three variations	**89.00**

## Data Availability

The CASIA b dataset was utilized for the experimental process. This dataset is publically available for research purposes at the Center for Biometrics and Security Research (ia.ac.cn).
